# Methylene blue reduces the serum levels of interleukin-6 and inhibits STAT3 activation in the brain and the skin of lipopolysaccharide-administered mice

**DOI:** 10.3389/fimmu.2023.1181932

**Published:** 2023-05-30

**Authors:** Yujia Li, Weihai Ying

**Affiliations:** Med-X Research Institute and School of Biomedical Engineering, Shanghai Jiao Tong University, Shanghai, China

**Keywords:** IL-6, STAT3, methylene blue, LPS, neuroinflammation

## Abstract

It is valuable to search for novel and economical agents for inhibiting STAT3 activation and blocking increases in IL-6 levels, due to the important roles of STAT3 and IL-6 in inflammation. Since Methylene Blue (MB) has shown therapeutical potential for multiple diseases, it has become increasingly important to investigate the mechanisms underlying the effects of MB on inflammation. Using a mouse model of lipopolysaccharide (LPS)-induced inflammation, we investigated the mechanisms underlying the effects of MB on inflammation, obtaining the following findings: First, MB administration attenuated the LPS-induced increases in the serum levels of IL-6; second, MB administration attenuated LPS-induced STAT3 activation of the brain; and third, MB administration attenuated LPS-induced STAT3 activation of the skin. Collectively, our study has suggested that MB administration can decrease the levels of IL-6 and STAT3 activation - two important factors in inflammation. Since MB is a clinically used and relatively economical drug, our findings have suggested therapeutic potential of MB for multiple inflammation-associated diseases due to its effects on STAT3 activation and IL-6 levels.

## 1 Introduction

Inflammation plays a critical role in numerous diseases (1–3). Cytokines such as IL-6 mediate multiple important inflammatory processes such as cytokine storm and fever (4–6). Therefore, it is significant to discover new agents that can effectively modulate the levels of cytokines and other key components in the inflammatory processes. IL-6 plays crucial roles in multiple pathological processes including cytokine storm that mediates pathological processes such as COVID-19-induced syndromes and fever (4–6). The biological effects of IL-6 are mediated *via* the gp130 cytokine receptor family, stimulation of which leads to activation of the Janus kinase-signal transducer and activator of transcription (JAK-STAT) signaling cascade. It is established that IL-6 acts through the STAT3 isoform that is phosphorylated, dimerized, and subsequently translocated into the nucleus, leading to the regulation of gene expression by binding to specific gene promoters (7). To inhibit IL-6-mediated inflammatory processes, broad-spectrum anti-inflammatory drugs (8) as well as agents targeting at IL-6 signaling pathways (9–13) have been used under clinical conditions. However, these drugs and antibodies have significant clinical limitations and problems (14–17).

STAT3 is a key factor in inflammation, which plays critical roles in multiple diseases including cancer development (18). STAT3 activation can be induced by such factors including IL-6 and IL-10, which can induce multiple inflammatory reactions (19). A large number of studies have indicated the critical significance of STAT3 activation as a mediator of inflammation and immunity (20) (1): As a key convergence of several signaling pathways, STAT3 plays critical roles in several biological processes including inflammatory responses (21) (2); hyper-activation of NF-κB is induced by synergistic interactions between NF-κB and STAT3, leading to production of various inflammatory cytokines (22) (3); the transcription factors nuclear factor-κB (NF-κB) and STAT3 play central roles in inflammation-mediated tumor promotion and metastasis (23); and (4) STAT3 activation plays critical roles in a number of inflammatory processes (24–31). It is warranted to discover new and economic agents targeting at STAT3.

Methylene Blue (MB) is the first fully synthetic drug for treating malaria (32), which has been used clinically both in the diagnosis and treatment of diseases: MB is used in the detection of leaks or positions of parathyroid corpuscles during surgery; and MB has been used for treating multiple diseases including methemoglobinemia, vasoplegic syndrome, cyanide intoxication, and ifosfamide-induced encephalopathy (33). Recently there was an increasing interest in MB as an antimalarial agent and potential therapeutic agent for neurodegenerative diseases (34). One of the major mechanisms underlying the beneficial effects of MB is the capacity of MB to decrease the production of reactive oxygen species (ROS) from the mitochondrial electron transport chain (35, 36). Therefore, MB is also used as one of the mitochondrial-targeted antioxidants (37). Increasing evidence has suggested that MB can also produce beneficial effects by its anti-inflammation capacity: MB can decrease lung injury in a rat model of hind limb ischemia-reperfusion by decreasing inflammation (38); MB can also attenuate ischemia-reperfusion injury in a rat model of lung transplantation by decreasing several indices of inflammation (39).

It becomes increasingly important to conduct studies to further investigate the effects of MB on inflammation and to investigate the mechanisms underlying the effects of MB on inflammation, due to the following two reasons: First, there has been no comprehensive study and understanding on the effects of MB on systemic inflammation, neuroinflammation and local inflammation in other tissues and organs; and second, the mechanisms underlying the effects of MB on inflammation have been unclear.

In this study, we determined the effects of MB administration on the neuroinflammation and skin inflammation in LPS-administered mice. LPS, the outer membrane of gram-negative bacteria, is a ligand of Toll-like receptor 4 (TLR4), which has been widely used to initiate inflammatory responses in multiple organs and tissues including the CNS (40). According to the comprehensive reviews on animal models of LPS-induced neuroinflammation, the major hallmarks for determinations of LPS-induced neuroinflammation include (41, 42) (1): Microglial activation – a key event in neuroinflammation, which has been the main outcome parameter in all examined studies (41). Microglial activation has been most widely determined by determinations of the levels of ionized calcium-binding adapter molecule 1 (Iba-1) – a biomarker for microglial activation (41) (2); the protein levels of iNOS and COX2 – Two pro-inflammatory effector enzymes - are induced and up-regulated upon inflammation (42) (3); determinations of the mRNA levels of cytokines including IL-1β, IL-6 and TNF-α (42); and (4) determinations of cognitive dysfunction and weight loss (41, 43). A number of *in vivo* and *in vitro* studies have also shown that LPS can induce inflammatory responses in the skin tissue and skin cell cultures (44, 45), e.g. it was reported that LPS-induced skin edema and neutrophil recruitment in mice (44).

Using a mouse model of LPS-induced inflammation, our study was designed to obtain the information mentioned above. In this model, LPS can induce systemic inflammation, neuroinflammation, and local inflammation in other tissues and organs (42). Our study has found that MB attenuated the LPS-induced increases in the serum levels of IL-6 levels; and in both the CNS and the skin, MB significantly attenuated LPS-induced STAT3 activation.

## 2 Materials and methods

### 2.1 Reagents

LPS (L4130), and Methylene Blue (66720) were purchased from Sigma Aldrich (St Louis, MO, United States).

### 2.2 Animal studies

The Ethics Committee of Animal Study of the School of Biomedical Engineering, Shanghai Jiao Tong University granted approval for the animal research protocol used in this study (Approval#: 2021010). The mice used in this study were male C57BL/6 mice aged 5–6 weeks, purchased from Shanghai Lingchang Laboratory Animal Inc. (Shanghai, China). Mice were kept in a 22°C environment with 12 hours of light and 12 hours of darkness each day, with free access to food and water. The mice were divided randomly into four groups after 2 weeks (1): The control group: The mice were administered with PBS intraperitoneally (i.p.) every day for 3 days (2). The 10 mg/kg MB treatment group: The mice received intraperitoneal injection of MB (5 mg/kg body weight) dissolved in PBS for 3 days (3). The 20 mg/kg MB treatment group: The mice received intraperitoneal injection of MB (20 mg/kg body weight) dissolved in PBS for 3 days (4). The LPS group: The mice were administered with LPS (1.0 mg/kg body weight) intraperitoneally (i.p.) every day for 3 days. LPS dissolved in PBS (5). The low-dose MB group: The mice received intraperitoneal injection of MB (5 mg/kg body weight) dissolved in PBS at 0.5 hours following LPS for 3 days (6). The high-dose MB group: The mice received intraperitoneal injection of MB (10 mg/kg body weight) dissolved in PBS at 0.5 hours following LPS for 3 days. Then tribromoethanol was given intraperitoneally to the mice. Serum was collected, and the mice were perfused with PBS and 4% paraformaldehyde (PFA) solution (for immunofluorescence staining assay). For Western blot experiments, the skin tissues collected from the ears of the mice were flash-frozen, and the brain tissues were either removed or sliced with a cryostat to a 30-μm thickness.

### 2.3 Animal body weight

Animals were weighed before LPS/MB administration (T=0 day) and at 1, 2 and 3 days post-LPS/MB injection.

### 2.4 Determinations of the serum levels of cytokine

A Bio-Plex Pro test kit was used to measure the mice’s serum concentrations of a number of cytokines (Bio-Rad Laboratories). The assay plate received a total of 50 μl of antibody-conjugated beads. 50 μl of diluted samples, the blank, standards, and the controls were applied to the plate after the samples had been diluted by 1:4. The plate was shaken vigorously at 300 rpm for 30 minutes while being incubated at room temperature (RT) in the dark. The plate received a total of 25 μl biotinylated antibody after three washes with 100 μl wash buffer. The plate was shaken vigorously at 300 rpm for 30 minutes in the dark at RT with 100 μl washing buffer 3 times. The plate was then filled with a total of 50 μl streptavidin-phycoerythrin (PE) and incubated for 10 minutes in the dark at RT with 300 rpm shaking. A Bio-Plex protein array reader evaluated the samples after three washes. Data collection and assay analysis were done using Bio-Plex Manager 6.0 software.

### 2.5 Western blot assay

The mice’s cortex, hippocampus, or skin were placed in the microcentrifuge tube on ice. Add 100 μl of pre-cooled RIPA buffer (Millipore, Temecula, California, United States) per 10 mg of tissue and homogenize in an ice bath using a tissue homogenizer. The RIPA buffer was added with a 1% protease inhibitor cocktail, 1% phosphatase inhibitor cocktail (CWBio, Beijing, China), and 1 mM phenylmethanesulfonyl fluoride before use. Gently aspirate the supernatant and transfer it to a newly prepared, pre-cooled microcentrifuge tube on ice after centrifuging at 4°C for 20 minutes at 12000 rpm. Following the BCA Protein Assay Kit’s measurement of the protein samples (Pierce Biotechnology, Rockford, Illinois, United States), 30 μg of total protein were added to 10% sodium dodecyl sulfate-polyacrylamide gel for electrophoresis and then transferred to 0.45-μm nitrocellulose membranes. Blocking the blots with 5% skimmed milk for 1 hour at RT. After 3 washes with TBST buffer, primer antibodies were incubated with the blots for a whole night at 4°C. The antibody dilutions were as follows: p-STAT3 antibody (1:2,000 dilution, Cell Signaling Technology, Danvers, MA), STAT3 antibody (1:2,000 dilution, Cell Signaling Technology, Danvers, MA), iNOS antibody (1:1,000 dilution, Abcam, Cambridge, United States), COX2 antibody (1:800 dilution, Abcam, Cambridge, United States) and Actin antibody (1:1,000 dilution, Santa Cruz, California, United States). After that, the blots were incubated with HRP conjugated Goat Anti-Rabbit IgG (H+L) (1:4000, Jackson ImmunoResearch, PA, USA) or HRP conjugated Goat Anti-mouse IgG (1:4000, Jackson ImmunoResearch, PA, USA). The protein signals were found using an ECL detection system (Thermo Scientific, Pierce, IL, USA). Densitometry was used with Image J to quantify the band intensities.

### 2.6 Immunofluorescence staining

Cryosections of the brain were first fixed in 4% PFA for 15 min, then incubated in 0.2% Triton X-100 in PBS for 10 minutes. Blocking the sections with 10% goat serum for an hour at RT and then Iba-1 antibody (1:500 dilution, Cell Signaling Technology, MA, USA) was incubated on the sections overnight at 4°C after three PBS washes. Following three PBS washes, the sections were incubated for 1 hour at RT with Alexa Fluor^®^ 488 goat anti-rabbit IgG (H + L) Secondary antibody (1:500 dilution, Molecular Probes, Eugene, Oregon, United States) in PBS containing 1% goat serum. The sections underwent three PBS washes before being counterstained with DAPI (1:1,000 dilution, Beyotime Institute of Biotechnology) in PBS for 5 min at RT. A Leica confocal microscope (Leica Microsystems, Wetzlar, Germany) was used to capture the fluorescence pictures, which were then processed by ImageJ.

### 2.7 Statistical analyses

All data are displayed as mean ± SEM. One-way ANOVA was used to analyze the data, and then a Student-Newman-Keuls *post hoc* test was performed. Statistics were considered significant for *P* values less than 0.05.

## 3 Results

### 3.1 MB attenuated LPS-induced increases in the serum levels of IL-6 in LPS-administered mice

**Figure 1:**
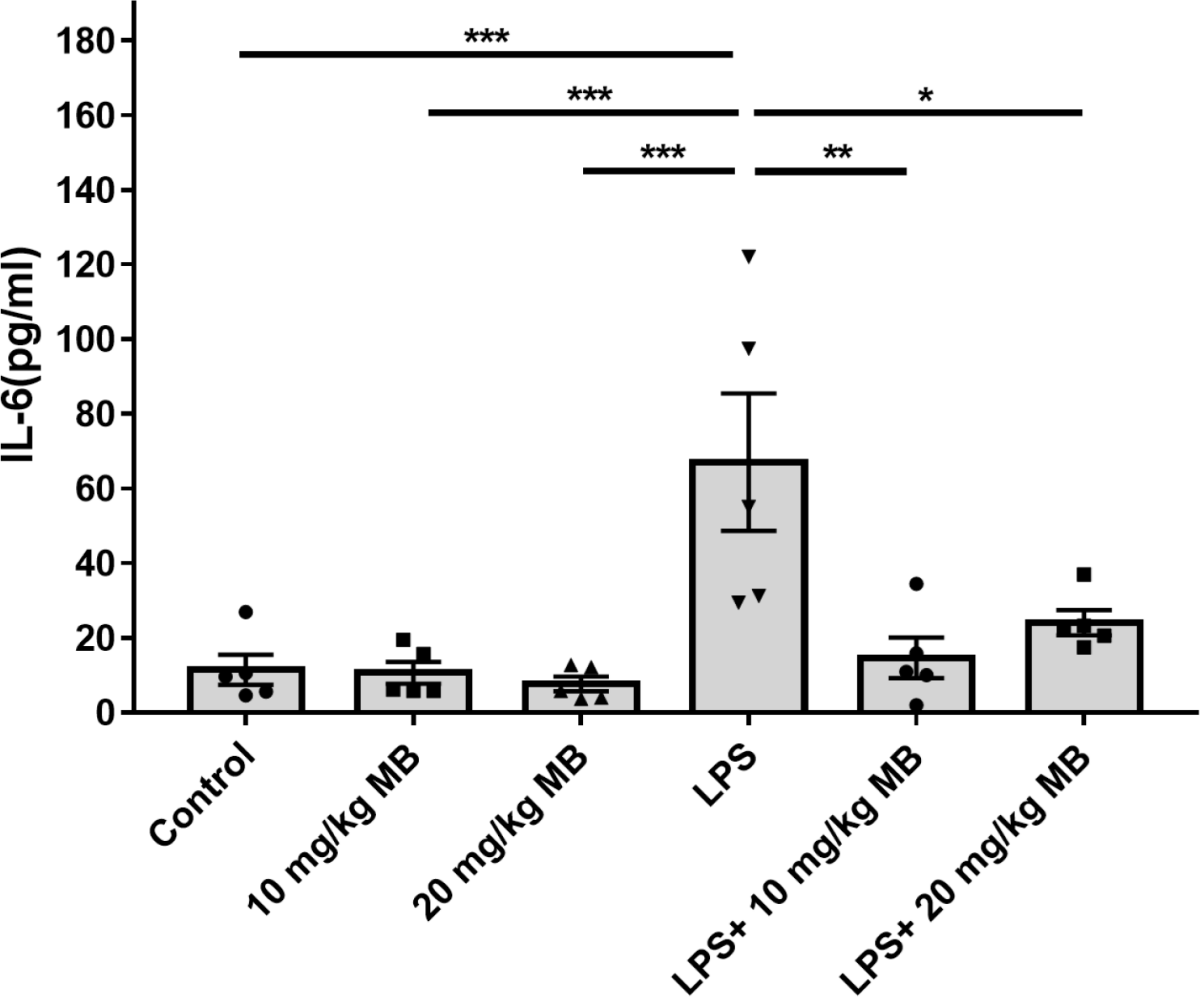
MB selectively attenuated LPS-induced increases in the serum levels of IL-6 levels of the mice. Three days after the administration of 1.0 mg/kg LPS, the serum levels of multiple cytokines were determined. N =5; *, *P* < 0.05; **, *P* < 0.01; ***, *P* < 0.001.

We determined the levels of a number of cytokines in the serum of LPS-administered mice three days after the mice were administered with LPS: LPS induced significant increases in the serum levels of multiple cytokines, including IL-6 (**Figure 1**), IL-1α (**Supplemental Figure 1A**), IL-1β (**Supplemental Figure 1B**), IL-10 (**Supplemental Figure 1C**), G-CSF (**Supplemental Figure 1E**), MCP-1 (**Supplemental Figure 1F**), MIP-1α (**Supplemental Figure 1G**) and MIP-1β (**Supplemental Figure 1H**). In contrast, In contrast, LPS induced a significant decrease in the serum level of IL-12(p40) (**Supplemental Figure 1D**). LPS did not significantly increase the serum levels of IL-2, IL-3, IL-4, IL-5, IL-9, IL-12(p70), IL-13 IL-17A, Eotaxin, GM-CSF, IFN-γ, KC, RANTES and TNF-α (**Supplemental Table 1**). It is noteworthy that the LPS-induced increases in the serum level of IL-6 were significantly attenuated by both 10 and 20 mg/kg MB in the mice (**Figure 1**).

### 3.2 MB administration decreased LPS-induced STAT3 activation in the brain of LPS-administered mice

Because IL-6 is an NF-κB target, simultaneous activation of NF-κB and STAT3 in non-immune cells triggers a positive feedback loop of NF-κB activation by the IL-6-STAT3 axis (22). A large number of studies have indicated that IL-6 is upstream of STAT3 activation, which forms IL-6-STAT3 axis in inflammation (22, 46). Therefore, we proposed our hypothesis that the MB-induced reductions of the serum level of IL-6 might lead to decreased STAT3 activation in organs and tissues in LPS-administered mice.

**Figure 2:**
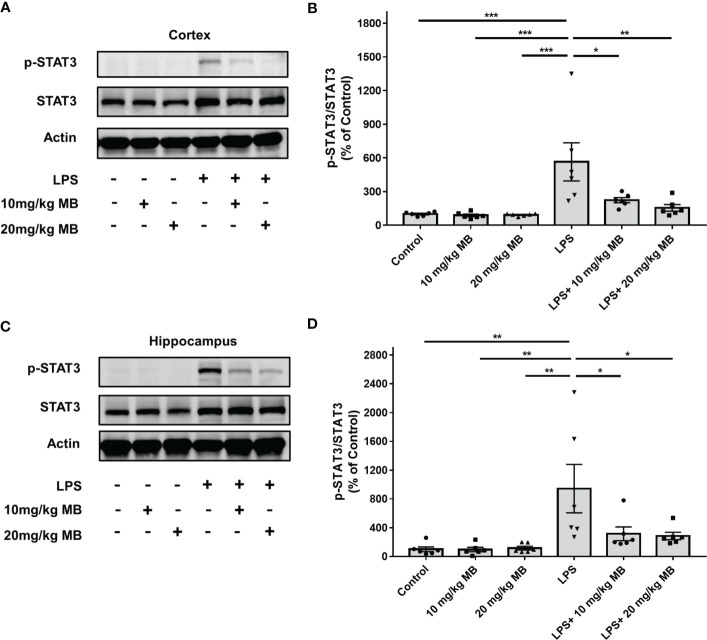
MB significantly attenuated LPS-induced increases in the ratios of pSTAT3/STAT3 in both the cerebral cortex and hippocampus of C57BL/6 mice. **(A)** LPS induced a significant increase in the ratio of pSTAT3/STAT3 in the cerebral cortex, which was significantly attenuated by MB administration. **(B)** Quantifications of the Western blots showed that MB significantly decreased the ratio of pSTAT3/STAT3 in the cerebral cortex of the LPS-administered mice. **(C)** LPS induced a significant increase in the ratio of pSTAT3/STAT3 in the hippocampus, which was significantly attenuated by MB administration. **(D)** Quantifications of the Western blots showed that MB significantly attenuated the ratio of pSTAT3/STAT3 in the hippocampus of the LPS-administered mice. Three days after the administration of 1.0 mg/kg LPS, Western blot assays on the brain tissues were conducted. N = 6; *, *P* < 0.05; **, *P* < 0.01; ***, *P* < 0.001. The data were representatives of three independent experiments.

We first determined the effects of MB on the levels of both STAT3 and the active form of STAT3 - phosphorylated STAT3 (pSTAT3) (19) in both cortex and hippocampus of the CNS of LPS-administered mice: LPS induced a significant increase in the ratio of pSTAT3/STAT3 - an index of STAT3 activation - in the cortex, which was profoundly attenuated by both 10 and 20 mg/kg MB (**Figures 2A, B**). Similarly, LPS also induced a significant increase in the ratio of pSTAT3/STAT3 in the hippocampus, which was profoundly attenuated by both 10 and 20 mg/kg MB (**Figures 2C, D**).

**Figure 3:**
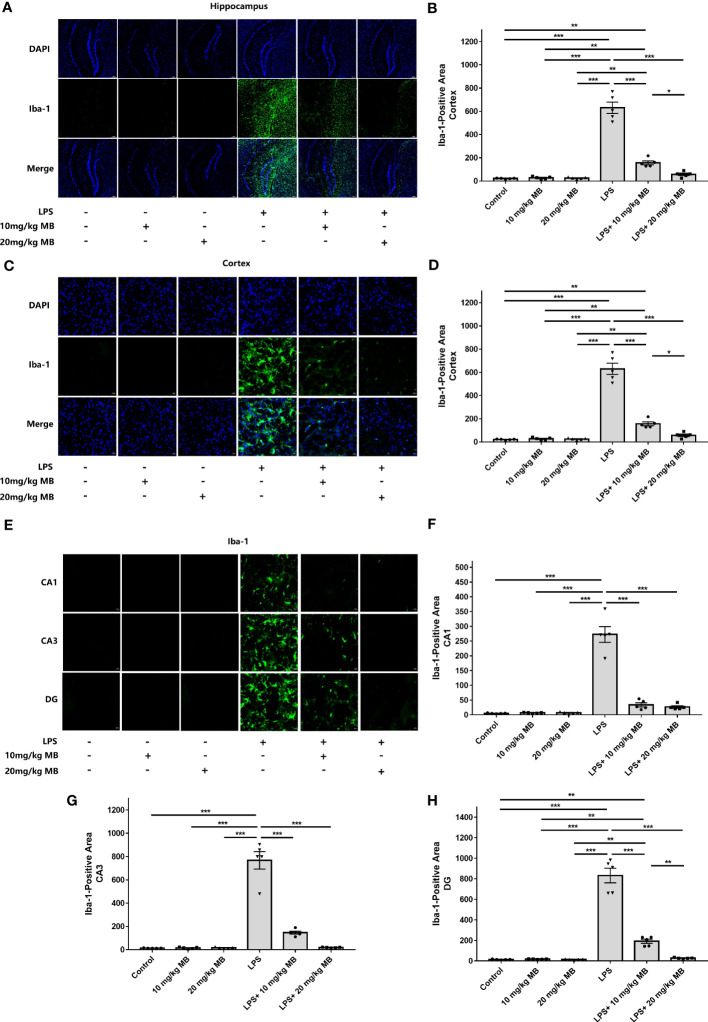
MB significantly attenuated LPS-induced increases in microglial activation in both the cerebral cortex and hippocampus of C57BL/6 mice. **(A)** The immunofluorescence staining assays showed that LPS induced a significant increase in the levels of Iba-1 – a hallmark of microglial activation - in the cerebral cortex of the mice, which was reversed by MB administration. **(B)** Quantifications of the images indicated that MB significantly attenuated LPS-induced Iba-1 increases in the cerebral cortex of the mice. **(C)** The immunofluorescence staining assays showed that LPS induced a significant increase in the levels of Iba-1 in the hippocampus of the mice, which was reversed by MB administration. **(D)** Quantifications of the images indicated that MB significantly attenuated LPS-induced Iba-1 increases in the hippocampus of the mice. **(E)** The immunofluorescence staining assays showed that MB significantly attenuated LPS-induced Iba-1 increases in the hippocampal CA1, CA3, and Dente Gyrus areas of the mice, which were significantly attenuated by MB administration. **(F–H)** Quantifications of the images indicated that MB significantly attenuated LPS-induced Iba-1 increases in the hippocampal CA1, CA3, and DG areas of the mice. Three days after the administration of 1.0 mg/kg LPS, immunofluorescence staining assays on the brain tissues were conducted. For each animal, three slices of the brain were used for the analyses. N = 5; *, *P* < 0.05; **, *P* < 0.01; ***, *P* < 0.001. The data were representatives of two independent experiments.

Since previous studies have indicated the critical roles of STAT3 activation in various types of neuroinflammation including LPS-induced neuroinflammation (24–28, 30, 31), we proposed the hypothesis that MB may decrease LPS-induced neuroinflammation. Therefore, we determined the effects of MB on various major indices of neuroinflammation: We found that LPS induced a significant increase in the levels of Iba-1 - a marker of microglial activation (47) - in the cortex, which was blocked by MB administration (**Figures 3A, B**). LPS also induced an increase in the levels of Iba-1 in the hippocampus, which was also blocked by MB administration (**Figures 3C, D**). We further determined the Iba-1 levels in the CA1, CA3, and Dente Gyrus, showing that LPS induced increases in the Iba-1 levels in all of the examined regions, which were attenuated by MB administration (**Figures 3E–H**).

**Figure 4:**
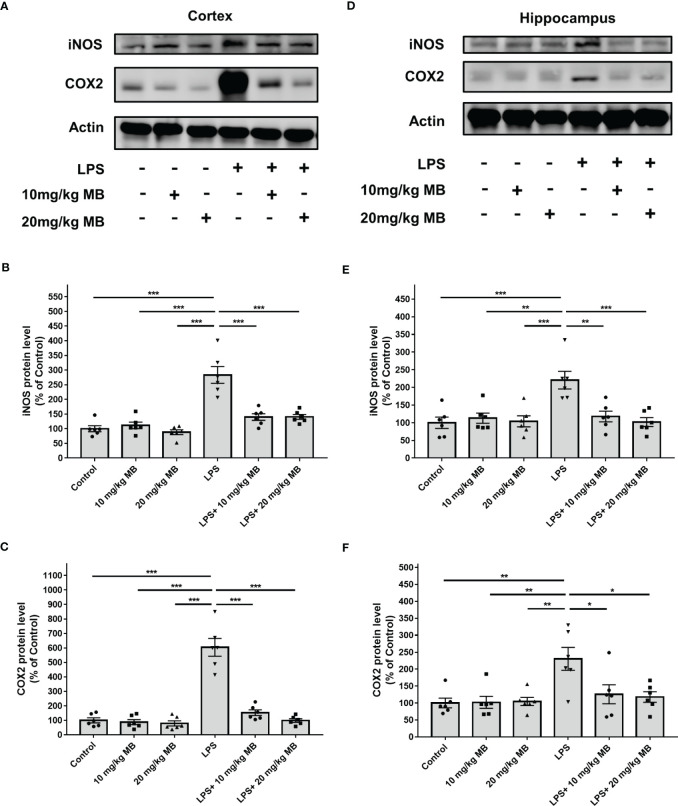
MB significantly attenuated LPS-induced increases in both iNOS and COX2 levels in the cerebral cortex and hippocampus of C57BL/6 mice. **(A)** Western blot assays showed that LPS induced significant increases in both iNOS and COX2 levels in the cerebral cortex of mice, which were significantly attenuated by MB administration. **(B, C)** Quantifications of the Western blots showed that MB significantly attenuated the LPS-induced increases in both iNOS and COX2 levels in the cerebral cortex. **(D)** Western blot assays showed that LPS induced significant increases in both iNOS and COX2 levels in the hippocampus of mice, which were significantly attenuated by MB administration. **(E, F)** Quantifications of the Western blots showed that MB significantly attenuated the LPS-induced increases in both iNOS and COX2 levels in the hippocampus. Three days after the administration of 1.0 mg/kg LPS, Western blot assays on the brain tissues were conducted. N =6; *, *P* < 0.05; **, *P* < 0.01; ***, *P* < 0.001. The data were representatives of three independent experiments.

Increased levels of inducible nitric oxide synthase (iNOS) and cyclooxygenase (COX)-2 are major indices of inflammation (48). Our Western blot experiments revealed that LPS increased the levels of iNOS and COX2 in the cortex of LPS-administered mice, which were significantly attenuated by MB (**Figures 4A–C**). Our Western blot experiments further showed that LPS increased the level of iNOS and COX2 in the hippocampus of LPS-administered mice, which were significantly attenuated by MB (**Figures 4D–F**). We have also conducted a study to determine the effects of MB administration on the LPS-induced weight loss of the mice. We found that at 1, 2 or 3 days after LPS administration, the mice had significant weight loss, which was significantly attenuated by the administration of MB (**Supplemental Figure 2**).

### 3.3 MB administration significantly decreased STAT3 activation in the skin of LPS-administered mice

**Figure 5:**
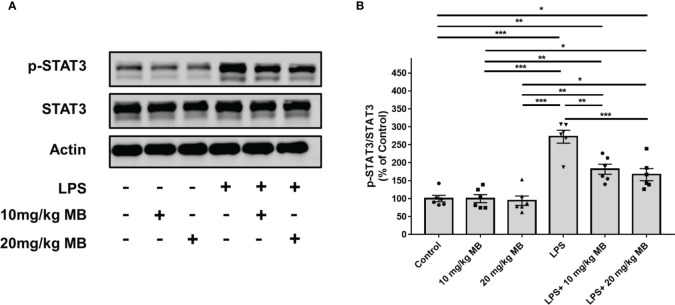
MB significantly decreased LPS-induced activation of STAT3 in the skin tissues collected from the ears of the mice. **(A)** LPS induced a significant increase in the ratio of pSTAT3/STAT3 in the skin of the mice, which was significantly attenuated by MB administration. **(B)** Quantifications of the Western blots showed that MB significantly attenuated the ratio of pSTAT3/STAT3 in the skin. Three days after the administration of 1.0 mg/kg LPS, Western blot assays on the skin tissues collected from the ears of the mice were conducted. N = 6; *, *P* < 0.05; **, *P* < 0.01; ***, *P* < 0.001. The data were representatives of two independent experiments.

**Figure 6:**
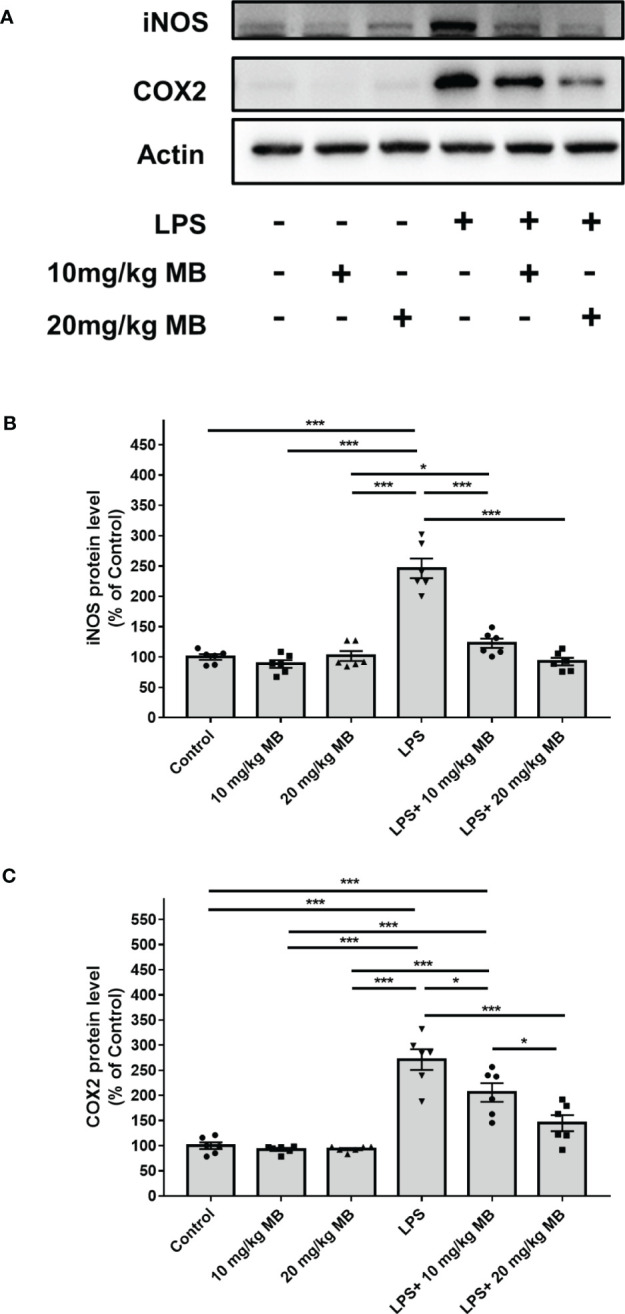
MB significantly attenuated LPS-induced increases in the iNOS and COX2 levels in the skin tissues collected from the ears of the mice. **(A)** Western blot assays showed that LPS induced a significant increase in the iNOS and COX2 levels in the skin of mice, which was significantly attenuated by MB administration. **(B, C)** Quantifications of the Western blots showed that MB significantly attenuated the LPS-induced increase in the iNOS and COX2 levels in the skin. Three days after the administration of 1.0 mg/kg LPS, Western blot assays on the skin tissues collected from the ears of the mice were conducted. N = 6; *, *P* < 0.05; ***, *P* < 0.001. The data were representatives of two independent experiments.

Since a large number of studies have indicated that IL-6 is upstream of STAT3 activation, which forms IL-6-STAT3 axis in inflammation (22, 46), we proposed the hypothesis that MB might also decrease STAT3 activation of the skin in LPS-administered mice. Therefore, we determined the effects of MB on the levels of pSTAT3 and STAT3 in the skin of LPS-administered mice: LPS induced a significant increase in the ratio of pSTAT3/STAT3 in the skin, which was significantly attenuated by MB administration (**Figures 5A, B**). We also found that LPS induced significant increases in the iNOS and COX2 levels in the skin, which were attenuated by MB administration (**Figures 6A–C**).

## 4 Discussion

MB is clinically used for the diagnosis and treatment of multiple diseases (33). Increasing evidence has also suggested the beneficial effects of MB in multiple animal models of diseases (38, 39). The antioxidant is an important mechanism for the beneficial effects of MB (35, 36), while there is evidence suggesting that anti-inflammation is also a mechanism (38, 39). It becomes increasingly important to conduct comprehensive studies on the effects of MB on systemic inflammation, neuroinflammation as well as the inflammation of other tissues and organs, and to investigate the mechanisms underlying the effects of MB on inflammation. Our current study has obtained new findings on these two important topics: Our study has shown that MB can not only decrease the serum level of IL-6, but also inhibit the activation of STAT3 in LPS-administered mice. It is established that STAT3 activation is the major effector of IL-6 (7), and both STAT3 activation (24–31) and IL-6 (4–6) play critical roles in inflammation. Therefore, our findings indicating the capacity of MB in decreasing both STAT3 activation and the serum levels of IL-6 in LPS-administered mice have suggested important mechanisms underlying the effects of MB on inflammation. Since aging is a critical risk factor for multiple major diseases, the accelerated aging around the world has led to significant increases in the number of patients with multiple age-related diseases, many of which are related to inflammation (1–3, 49). Economical diagnosis and treatment are highly needed. Therefore, MB may become a relatively economical agent for treating diseases.

On the basis the well-established criteria for determinations of LPS-induced neuroinflammation (41, 42), our study has provided four lines of evidence indicating that MB can significantly attenuate LPS-induced neuroinflammation (1): By using immunostaining assays, our study determined the changes of Iba-1 levels which indicate microglia activation (41) in the cortex and the hippocampus. Our study has provided solid evidence indicating that LPS induced significant increases in Iba-1 levels, which were significantly attenuated by MB administration (**Figure 3**), indicating that MB can significantly decrease LPS-induced microglial activation (2). Our study has shown that MB administration significantly attenuated LPS-induced increases in the protein levels of two pro-inflammatory effector enzymes, iNOS and COX2 (**Figure 4**) (42), which has further indicated that MB can significantly decrease LPS-induced neuroinflammation (3). Our study has found that MB can significantly attenuate LPS-induced STAT3 activation. A large number of studies have indicated that STAT3 activation, like nuclear NF-kB translocation, is a mediating factor in inflammation, which plays a key role in inflammatory processes (20–23). Therefore, it is reasonable for us to propose that MB may decrease LPS-induced microglial activation at least partially by attenuating LPS-induced STAT3 activation (4). Our study has also determined the effects of MB administration on LPS-induced weight loss of mice, showing that MB administration significantly attenuated the LPS-induced weight loss of the mice. It is warranted to conduct future studies to further investigate the mechanisms underlying these observations.

Our study has also provided two lines of evidence indicating that MB administration can significantly decrease LPS-induced skin inflammation of the mice (1): iNOS and COX2 are two pro-inflammatory effector enzymes (42), which are also hallmarks of skin inflammation (50, 51). Our study found that MB significantly attenuated LPS-induced increase in both iNOS and COX2 levels, which were significantly attenuated by the MB administration (2). STAT3 activation has been indicated as an important mediator of inflammation, which is a key pathological factor in inflammatory processes (22, 52). Our study found that LPS induced a significant increase in STAT3 activation in the skin, which was significantly attenuated by the MB administration.

Our finding that MB can attenuate the increases in the serum levels of IL-6 in LPS-administered mice has indicated the therapeutic potential of MB for multiple diseases: Cumulative evidence has indicated that increased IL-6 is a key factor initiating cytokine storm, which plays crucial roles in such pathology as COVID-19-induced severe syndrome (22, 53). The current strategy for inhibiting cytokine storm includes the administration of broad-spectrum anti-inflammation drugs such as glucocorticoids (8) or administration of agents that block the IL-6 signaling pathway, including IL-6 receptor antagonists such as Tocilizumab (9), antibodies against IL-6 such as Siltuximab (10), and antibodies blocking the IL-6 receptor such as Sarilumab (11–13). However, the major problem for the administration of broad-spectrum anti-inflammation drugs is the significant side effects of the drugs such as hyperglycemia, hypertension, and osteoporosis (14, 15), while the major problems for the administration of the agents targeting at IL-6 signaling pathways include serious infection (16), loss of efficacy and/or immune-mediated adverse reactions (17) and relatively high cost of the drug. MB is a clinically used and relatively economical agent, which holds the potential to become a drug for inhibiting cytokine storms. It is necessary to conduct future studies to determine the pathological conditions under which MB can significantly decrease the serum level of IL-6 and to search for the mechanisms underlying the MB-produced reductions of the increased serum levels of IL-6.

Microglial activation has been most widely determined by determinations of the levels of Iba-1 – a hallmark for microglial activation (41). Expression of Iba1 is up-regulated in activated microglia in the CNS under inflammatory conditions in multiple neurological diseases such as Alzheimer’s disease (54) and traumatic brain injury (55), which have indicated that Iba-1 is an activated phenotype of microglia. Iba-1 has been reported to be a key molecule in membrane ruffling and phagocytosis (56). Based on the previous reports regarding the biological functions of Iba-1, it is expected that the Iba-1 increases may significantly contribute to microglial activation by promoting such processes as phagocytosis. Therefore, future studies are warranted to determine the effects of certain drugs targeting at the increased Iba-1 under inflammatory conditions. This strategy might be valuable for decreasing the neuroinflammation in such diseases as brain ischemia and Parkinson disease.

IL-12 is a pro-inflammatory cytokine that forms a link between innate resistance and adaptive immunity (57). The cytokine favors the differentiation of T helper 1 (T(H)1) cells (57). In contrast to the other examined cytokines, our study showed that the serum level of IL-12(p40) was significantly decreased by LPS administration, which was not significantly affected by the MB administration (**Supplemental Figure 1D**). It is warranted to determine the mechanism underlying this observation.

MB has diverse applications and uses, such as its anti-malarial properties, its capacity to treat methemoglobinemia, and its effectiveness as a diagnostic tool. Nevertheless, MB may potentially cause adverse effects on the body: Excessive amounts of MB may lead to hypertension, increased vascular resistance, and arrhythmia and dysfunction in cardiac function (58). Therefore, it is essential to master the appropriate concentration of MB usage. Another negative consequence of MB usage is the potential to cause anemia in individuals with glucose-6-phosphate dehydrogenase deficiency. Additionally, infants exposed to MB may develop hyperbilirubinemia, ultimately leading to jaundice (59). The principal detrimental repercussion among adult patients pertains to the utilization of selective serotonin reuptake inhibitors (SSRIs), which are commonly administered psychiatric medicines. When co-administered with MB, a robust monoamine oxidase inhibitor (MAOI), they may trigger serotonin syndrome – a life-threatening medical emergency (60). Therefore, prior to the administration of MB, it is advisable to screen patients for the presence of these drugs, considering their widespread usage. Nevertheless, the reasonably minimal and foreseeable hazards associated with MB render it an appropriate option for implementation in clinical settings.

## Data availability statement

The original contributions presented in the study are included in the article/**Supplementary Material**. Further inquiries can be directed to the corresponding author.

## Ethics statement

The animal study was reviewed and approved by The Ethics Committee of Animal Study of the School of Biomedical Engineering, Shanghai Jiao Tong University.

## Author contributions

YL contributed to the conception and design of the study, analyses of the data, and writing of the article. WY directed the project and contributed to the writing of the article. All authors approved the submitted version. All authors contributed to the article and approved the submitted version.
