# Supplementary material for: Methylene blue reduces the serum levels of interleukin-6 and inhibits STAT3 activation in the brain and the skin of lipopolysaccharide-administered mice
Source: Front Immunol. 2023 May 30;14:1181932. doi: 10.3389/fimmu.2023.1181932 (PMC10266349; doi:10.3389/fimmu.2023.1181932)
Supplement: Supplementary file 1 [file DataSheet_1.docx]

Supplementary Material

Methylene Blue Reduces the Serum Levels of Interleukin-6 and Inhibits STAT3 Activation in Lipopolysaccharide-Administered Mice

Yujia Li, Weihai Ying*

*** Correspondence:** Weihai Ying: weihaiy@sjtu.edu.cn

# Supplementary Figure


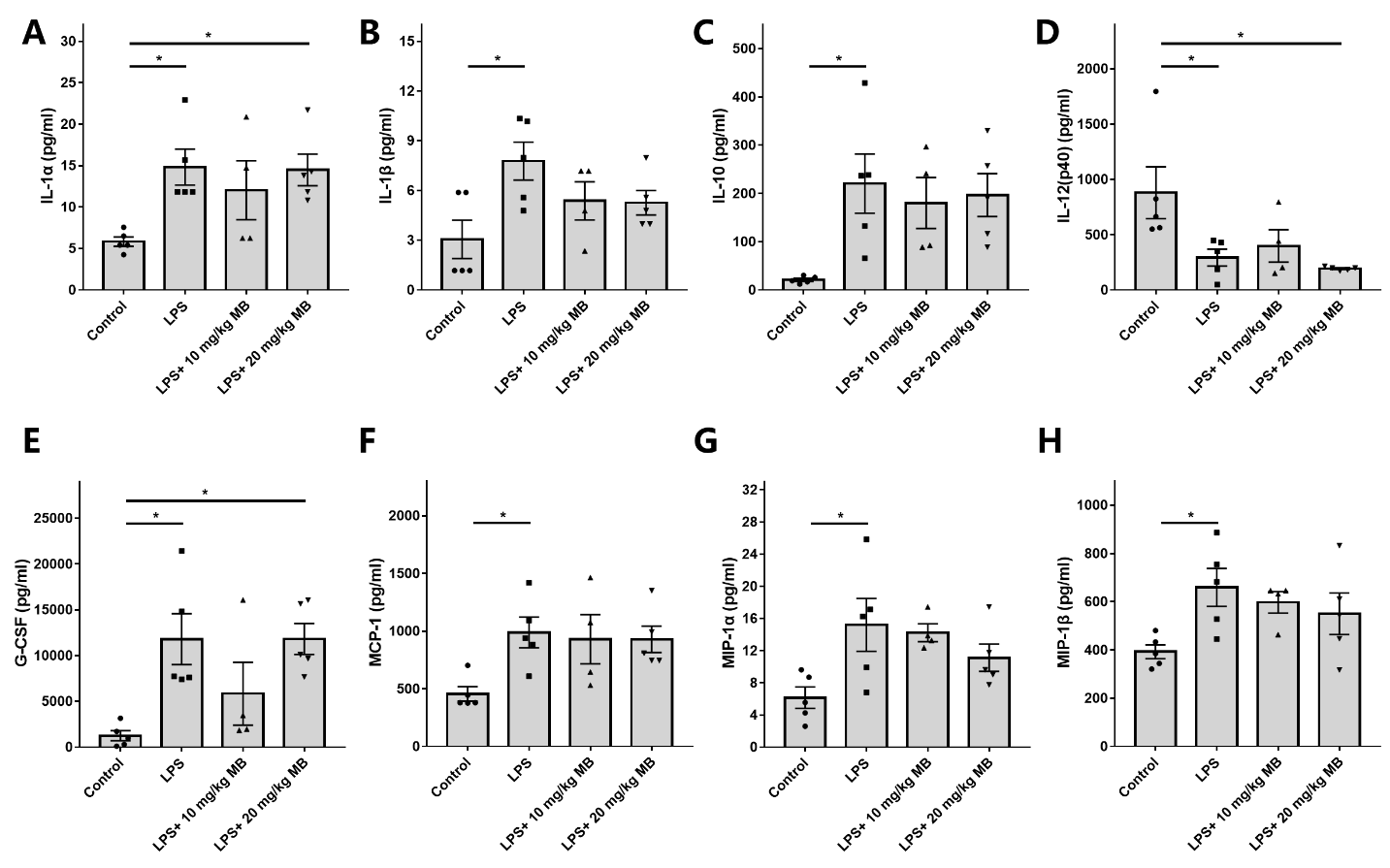


**Supplementary Figure 1.** LPS causes significant changes in multiple cytokine levels. (A, B, C, E, F, G) LPS induced significant increases in the serum levels of multiple cytokines, including IL-1α, IL-1β, IL-10, G-CSF, MCP-1, MIP-1α, and MIP-1β. (D) LPS induced significant decreases in the serum levels of IL-12 in the LPS-administered mice. Three days after the administration of 1.0 mg/kg LPS, the serum levels of multiple cytokines were determined. N =4-5; *, *P* < 0.05.


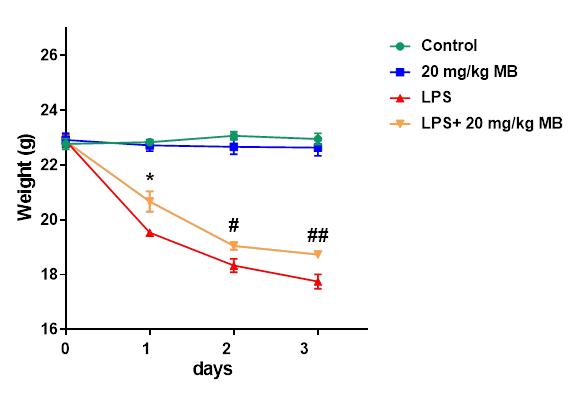


**Supplementary Figure 2.** MB significantly attenuated the LPS-induced weight loss of mice. Body weight was measured at 0, 1, 2 and 3 days after injections (PBS, 20 mg/kg MB, LPS, LPS+20 mg/kg MB). N = 6; *, *P* < 0.05; #, *P* < 0.05 (student t-test); ##, *P* < 0.05 (student t-test). The statistical symbol indicates the difference between the LPS group and the LPS+20 mg/kg MB Group.

**Supplementary Table 1.** LPS did not increase the serum levels of IL-2, IL-3, IL-4, IL-5, IL-9, IL-12(p70), IL-13 IL-17A, Eotaxin, GM-CSF, IFN-γ, KC, RANTES, TNF-α in LPS-administered mice. Three days after administration of 1.0 mg/kg LPS, the serum levels of the cytokines in the LPS-administered mice were determined. N =4-5.

| *pg/ml* | Control | LPS | LPS+10 mg/kg MB | LPS+20 mg/kg MB |
| --- | --- | --- | --- | --- |
| *IL-2* | 0.62±0.56 | 14.40±22.30 | 11.85±11.73 | 26.06±33.69 |
| *IL-3* | 2.40±0.67 | 1.94±1.94 | 1.96±0.81 | 1.66±1.21 |
| *IL-4* | 2.25±0.58 | 1.58±2.30 | 1.55±0.88 | 1.67±1.24 |
| *IL-5* | 8.91±6.37 | 14.61±8.26 | 6.35±0.00 | 17.90±0.00 |
| *IL-9* | 49.96±10.25 | 48.68±35.96 | 55.20±23.31 | 43.42±28.16 |
| *IL-12(p70)* | 179.64±29.55 | 121.40±122.16 | 129.93±80.09 | 107.26±86.89 |
| *IL-13* | 31.12±19.35 | 107.77±110.71 | 91.05±67.91 | 169.53±166.18 |
| *IL-17A* | 268.41±72.18 | 218.52±170.93 | 228.25±145.52 | 212.60±126.12 |
| *Eotaxin* | 1924.18±517.16 | 1740.15±571.90 | 1435.73±929.29 | 2255.89±771.91 |
| *GM-CSF* | 76.56±12.33 | 63.51±33.55 | 69.59±20.14 | 58.17±22.49 |
| *IFN-γ* | 30.69±6.24 | 26.75±23.33 | 31.90±17.45 | 24.96±18.20 |
| *KC* | 106.76±30.04 | 97.21±36.09 | 84.85±17.28 | 71.06±26.18 |
| *RANTES* | 206.21±57.24 | 470.64±164.41 | 503.95±328.63 | 581.63±137.90 |
| *TNF-α* | 87.23±21.91 | 91.80±80.58 | 103.13±61.99 | 76.01±51.94 |
